# The evolution of endobronchial ultrasound usage in modern era

**DOI:** 10.5578/tt.20239711

**Published:** 2023-09-22

**Authors:** A. GÜRÜN KAYA, D. DOĞAN

**Affiliations:** 1 Department of Chest Diseases, Ankara University Faculty of Medicine, Ankara, Türkiye; 2 Department of Chest Diseases, University of Health Sciences, Gülhane Faculty of Medicine, Ankara, Türkiye

**Keywords:** Endobronchial ultrasound, elastography, intranodal forceps biopsy, therapeutic application, convex probe ultrasound, cryobiopsy

## Abstract

**ABSTRACT:**

**The evolution of endobronchial ultrasound usage in modern era:**

Over the past two decades, endobronchial ultrasound (EBUS) has become a crucial tool for diagnosing pulmonary diseases. The most common indication of EBUS usage is the diagnosis and staging of lung cancer. Additionally, there have been significant improvements in the application of convex probe EBUS in line with the increase in experience and knowledge about EBUS and the developments in medicine and technology. To enhance diagnostic accuracy and acquire larger tissue samples, techniques such as cryo-biopsy guided by endobronchial ultrasound (EBUS) and intranodal forceps biopsy have been developed. Additionally, elastography functionality has been integrated into the EBUS application to improve the assessment of targeted lesions. Moreover, its utilization for evaluating and sampling pulmonary vascular structures has expanded. It has also found applications in guiding intratumoral therapy, positioning fiducial markers, and facilitating the drainage of pleural or pericardial effusions. This review aims to provide an overview of the extended applications of convex probe EBUS beyond its conventional uses.

## Abstract (tr)

**Modern çağda endobronşiyal ultrason kullanımının evrimi:**

Son yirmi yılda, endobronşiyal ultrasonografi (EBUS) akciğer hastalıklarının teşhisinde çok önemli bir araç haline gelmiştir. EBUS kullanımının en yaygın endikasyonu ise akciğer kanserinin tanısı ve evrelemesidir. Ayrıca, zaman içinde EBUS konusundaki tecrübe ve bilgi birikiminin artması, tıp ve teknolojideki gelişmelere paralel olarak konveks prob EBUS uygulamasında önemli gelişmelere olanak sağlamıştır. Daha iyi tanısal etkinlik sağlanması ve daha büyük bir örnek elde etmek için EBUS rehberliğinde uygulanan kriyo-biyopsi, intranodal forseps biyopsi gibi metotlar geliştirilmiştir. Ayrıca, hedeflenen lezyonların daha iyi değerlendirilmesi için elastografi işlevi EBUS uygulamasına entegre edilmiştir. Bunun yanı sıra pulmoner vasküler yapıların değerlendirilmesinde ve örneklenmesinde de EBUS kullanımı artmıştır. EBUS’un terapötik kullanım uygulamaları, intratümöral tedavi, referans işaretleyicinin yerleştirilmesi ve plevral veya perikardiyal efüzyonun boşaltılması için rehberlik olarak örneklenebilir. Bu rehberde konveks prob EBUS’un konvansiyonel kullanım alanlarının ötesinde farklı uygulama alanlarının özetlenmesi amaçlanmıştır.

The invention of the convex probe (CP) for endobronchial ultrasonography (EBUS) in 2002 marked a significant milestone in bronchoscopy. This technological advancement revolutionized the practice by enabling bronchoscopists to conduct real-time transbronchial needle aspiration (TBNA) procedures guided by EBUS. Over the last two decades, EBUS has undergone significant evolution and has emerged as a pivotal tool in the realm of pulmonary diseases. This technique allows bronchoscopists to visualize structures beyond the airways, including the airway wall, lung, and mediastinum, facilitating sample collection ( 1 , 2 ). Guidelines recommend EBUS as the primary approach for staging the mediastinum of lung cancer ( 3 , 4 ). Consistent with this, on a global scale, the primary applications of EBUS in general medical practice involve the identification of mediastinal and hilar lesions, as well as the staging of lymph nodes for lung cancer ( 5 ).

Over time, there has been a notable expansion in the clinical applications of this technique. The need for sufficient tissue for accurate cancer diagnosis and molecular analysis has led the bronchoscopist to modify their usual approach to ensure sample adequacy. Consequently, there has been a noticeable increase in the development of novel EBUS applications extending beyond the traditional endobronchial approach ( 1 , 1 , 1 ). This review aims to highlight the diverse applications of convex probe EBUS beyond its conventional role in diagnosing and staging lung cancer.

Innovative Methods for Obtaining Larger Tissue Samples Using Endobronchial Ultrasound

In recent years, personalized treatment plans have become crucial for patients with lung cancer. Different types of molecular tests are needed to guide these treatment plans, and obtaining sufficient material for cytological, immunohistochemical analysis, and molecular testing is essential to avoid repeat procedures ( 7 ). Additionally, the diagnostic yield of EBUS-TBNA is still debated in pulmonary pathologies other than lung cancer. Especially, for the diagnosis of lymphoproliferative diseases, larger tissue samples need to be acquired. Patients suspected of lymphoproliferative disease need to undergo mediastinoscopy despite benign cytological findings of EBUS-TBNA material ( 8 ). Furthermore, previous data suggested that the diagnostic accuracy of EBUSTBNA is still suboptimal, as it is unable to detect granulomas in around 20% of individuals diagnosed with sarcoidosis ( 9 , 10 ). Though mediastinoscopy is the gold standard for diagnosis, it is a relatively complex method with the potential for severe complications in some instances. Therefore, the search for new sampling strategies guided by EBUS has emerged ( 11 ).

CP-EBUS-Guided Transbronchial Mediastinal Cryo-Biopsy

To improve the collection of an adequate amount of histologically assessable material, a novel approach has recently been developed, which involves the integration of transbronchial mediastinal cryo-biopsy (TMC) with EBUS guidance. Using  that particular technique provides a greater quantity of mediastinal tissue for investigation compared  to needle aspiration  with minimal complication ( 12 ). In this technique, after the identification of the target lesion using the convex probe EBUS, its precise location, dimensions, and vascularization are documented. Subsequently, a high-frequency needle-knife is employed to create a small incision in the tracheobronchial wall near the mediastinal lesion. Following this, the knife is replaced by a cryoprobe connected to a cryo system, inserted into the working channel of the convex probe. A TBNA needle with repetitive punctures can be used in place of a knife for this step. Initiating the probe triggers a cooling process using liquid carbon dioxide, which is then retracted along with the bronchoscope, bringing the cryogenically frozen biopsy tissue with it ( 12 , 13 ).

Although its use has not become routine, the number of studies on EBUS-TMC applications has been increasing in recent years. The related studies and primary outcomes are presented in Table 1 . Furthermore, Oikonomidou et al. conducted a study to assess the size of the sampled lymph node tissue under the guidance of EBUS. They found that cellblock slices obtained using a 19G needle showed a higher count than those obtained with a 1.1 mm cryoprobe ( 14 ).

**Table 1:** Summary of studies related to transbronchial mediastinal cryo-biopsy by the guidance of convex probe endobronchial ultrasound

Study	Study group	Main outcome	Complications
Maturu et al. ( 7 )	46 patients, were subjected to EBUS-TMC due to *Non-diagnostic EBUS-TBNA (n= 32) *Inadequate material for EBUS-TBNA (n= 14)	*EBUS-TMC confirmed the diagnosis in 19 of 32 patients (59.2%) whose EBUS-TBNA non-diagnostic *All 14 patients with inadequate material by EBUS-TBNA obtained adequate material by EBUS-TMC	Minor bleeding in 13 patients
Fan et al. ( 16 )	271 patients were randomized into two groups: *Combination group EBUS-TBNA and EBUS-MCB (n= 136) *Control group; only EBUS-TBNA (n= 135)	The combined group had a higher diagnostic yield than the control group 94% vs. 81% risk ratio (RR) 1.15 (95% CI 1.04-1.26; p= 0.0039)	No severe complications causing death or disability. Airway bleeding combination group 2%, control group 1% [RR 0.67 (95% CI 0.11- 3.96); p= 1.00]
Zhang et al. ( 12 )	197 patients were allocated into two groups *“TBNA first” group (n= 99): TBNA followed by TMC *“Cryobiopsy first” group (n= 98): TMC followed by TBNA	The overall diagnostic yield was 79.9% and 91.8% for TBNA and transbronchial mediastinal cryo-biopsy, respectively (p= 0.001) Diagnostic yields were similar for metastatic lymphadenopathy (94.1% vs. 95.6%, p= 0.58), while cryo-biopsy was more sensitive than TBNA in uncommon tumors (91.7% vs. 25.0%, p= 0.001)	Four-week follow-up duration: *No major complications were noted *Most common adverse event: minor bleeding *Pneumothorax which resolved spontaneously at 1% *Pneumomediastinum, all of which resolved spontaneously at 0.5%
Ariza-Prota et al. ( 17 )	50 patients underwent EBUS-TBNA and EBUS-TMC in a single procedure.	*The diagnostic yield for EBUS-TBNA was 82%, and for EBUS-TMC 96% *TBNA diagnosed two patients that had an insufficient diagnosis by TMC *In nine cases, TBNA was not diagnostic; however, a definitive diagnosis was reached by cryo-biopsy TMC established the diagnosis of lymphoma in all patients in whom this pathology was suspected (100%) *The diagnostic concordance between the two techniques was 56%.	*Two-week follow-up duration: *No major complications *The most predominant adverse event was minor bleeding *No pneumothorax, pneumomediastinum or mediastinitis

While the idea of acquiring more substantial mediastinal specimens while preserving tissue architecture is appealing, there are also considerations associated with employing this technique. These include concerns about cost-effectiveness, whether it adds diagnostic value for specific molecular analyses

and PD-L1 expression, as well as its applicability to particular patient groups such as those with tuberculosis. In addition to these factors, interobserver variability is a significant concern that can potentially lead to discrepancies when interpreting the results of these studies ( 15 ).

EBUS Guided-Intranodal Forceps Biopsy

Intranodal forceps biopsy (IFB) has been developed to obtain a larger specimen volume for pathological evaluation. The EBUS-intranodal forceps biopsy (EBUS-IFB) procedure entails inserting micro forceps through the initial puncture site made by the TBNA needle. Previous studies have shown that combining intranodal forceps biopsy with EBUS-TBNA enhances the overall diagnostic yield ( 18 , 19 ).

The procedure requires a conventional EBUS bronchoscope, a TBNA needle, and mini forceps measuring 1.0 mm in diameter. The majority of research on EBUS-IFB procedures has utilized 22-gauge needles for TBNA. However, there are reports of successful outcomes utilizing EBUS-TBNA needles, including 19, 21, and even 25 gauge ( 20 ).

In the process of EBUS-TBNA, the airway mucosa is punctured four to five times using the EBUS-TBNA needle under ultrasound guidance. This results in a disruption of the mucous membrane of the airway, as well as the formation of a pathway through which mini forceps can be inserted into the specific lymph node being targeted. While ensuring the EBUS bronchoscope remains in a stable position, the procedure involves removing the aspiration needle from the working channel and subsequently inserting the mini forceps into the desired location, all under the guidance of EBUS. The direct view of the mucosal puncture site by endoscopy is frequently unattainable, making it an unreliable reference point for guiding the insertion of mini forceps ( 20 ).

Agrawal et al. performed a meta-analysis comprising six observational studies, which demonstrated that the use of EBUS-IFB combined with EBUS-TBNA enhances the overall diagnostic efficacy of intrathoracic adenopathy sampling in comparison to the use of EBUS-TBNA alone (92% vs. 67%; OR 5.87 95% confidence interval, 3.081 to 9.04; p< 0.001). The complication rates are comparatively higher in combination methods than those observed with EBUS-TBNA. However, they are purportedly reduced when compared to transbronchial or surgical biopsies. Moreover, the subgroup analysis revealed that the combination method had a higher diagnosis yield for lymphoma (86% vs. 30%; p= 0.03) and sarcoidosis (93% vs. 58%; p< 0.001) ( 21 ).

Another sampling instrument is the Acquire^®^ 22G fine needle, manufactured by Boston Scientific Co, which features a Franseen tip. The 22G fine needle instrument is designed with three cutting edges, allowing for the collection of larger specimens. Balwan et al. evaluated the feasibility and potential benefits of this approach in granulomatous disorders and determined a diagnostic yield of 95% (22). Furthermore, this needle can also be employed for obtaining samples from abdominal lesions guided by endoscopic ultrasound (23). Another tool is the ProCore® needle, developed by Cook Medical in Bloomington. It features a core trap mechanism designed specifically for obtaining histology samples. This is accomplished by shearing material from the lesion as the needle is in motion. The diagnostic accuracy of this needle appears to be comparable to that of a conventional 22-gauge needle in detecting both malignancy and sarcoidosis (24,25). The ProCore® needle is also used for extrathoracic lesion sampling. Karsenti et al. discovered that the Acquire needle can yield more tissue and exhibit improved diagnostic accuracy compared to the ProCore® needle when performing EBUS-guided biopsies of pancreatic masses ( 26 ).

Methods to Enhance Visualization Modalities in Endobronchial Ultrasound

Elastography

Elastography is a sensitive and accurate quantitative method for visualizing the distributions of strain and elastic modulus in soft tissues. Tissues exhibit a response to external or internal compression in the form of deformation, displacement, or velocity. This response can be digitally transmitted and overlaid into a 2-dimensional (2D) ultrasound image. The resulting image utilizes a color-coded system to represent tissue stiffness, with red indicating the softest, green indicating intermediate, and blue indicating the hardest stiffness levels. Malignant tumors possess a greater degree of stiffness compared to normal tissues and demonstrate reduced elasticity, hence enabling the potential of ultrasonic elastography to differentiate between malignant and benign lesions. Ultrasound elastography shows significant diagnostic efficacy in distinguishing between benign and malignant lesions  such as  the liver, kidney, pancreas, breast, thyroid, prostate, and lymph nodes. However, the ideal and established assessment approach for EBUS elastography remains uncertain, and there is a requirement for improvements in the performance of this technique as highlighted in much of the published research ( 5 , 27 , 28 ). In a metaanalysis including seventeen studies to investigate the impact of EBUS elastography for discrimination between benign and malignant hilar and mediastinal LNs., the authors showed the diagnostic performance of EBUS elastography for hilar and mediastinal lymph nodes is well-established, with a sensitivity of 0.90 (95% CI, 0.84-0.94) and a specificity of 0.78 (95% CI, 0.74-0.81) respectively ( 29 ). Concerning benign lesions, Madan et al. indicated that the diagnostic utility of elastographic lymph node features for distinguishing between tuberculosis and sarcoidosis during EBUS-TBNA is limited ( 30 ).

Visualization and sampling of vascular structures

EBUS enables notable visibility of the structures encompassing the airways, involving the vascular structures, including the aortic arch, left and right pulmonary artery trunk, azygos vein, and lobar arteries. In general practice, complete evaluation of those structures requires computed tomography and angiography. However, applying these techniques is restricted in patients with allergies, renal dysfunction, and during pregnancy due to the necessity of administering a contrast agent. In such scenarios, EBUS offers a diagnostic avenue, and several researchers have endeavored to utilize EBUS as a diagnostic tool for pulmonary vascular disease, involving both pulmonary embolism and nonembolic manifestations of the condition ( 31 , 32 , 33 , 34 ).

EBUS, equipped with color Doppler imaging capabilities, enables pulmonologists to rapidly evaluate the size of the thrombus and the extent of obstruction ( 6 ). The use of EBUS for the diagnosis of pulmonary embolism (PE) was first documented by Casoni et al. in a case involving a 26-year-old individual (33). Subsequently, other cases of pulmonary embolism identified by EBUS were reported ( 34 , 35 , 36 , 37 , 38 ). In 2017, Li et al. introduced a technique that provides guidance and orientation positions for exploring the left pulmonary artery, right pulmonary artery, and pulmonary trunk ( 39 ). A pilot study showed that EBUS has a detection accuracy of 96% in identifying PE, and this accuracy increases to 100% when exclusively focusing on centrally positioned emboli ( 40 ). Jull et al. conducted a study involving 100 patients suspected of having lung cancer, and their findings revealed a positive predictive value of 100% for EBUS in detecting pulmonary embolism ( 41 ).

EBUS not only allows pulmonary embolism but is also capable of detecting non-thrombotic pulmonary vascular pathologies, including vascular tumor invasion, tumor embolism, pulmonary arterial sarcoma, and arterio-venous malformation ( 1 , 1 , 1 ). Ghattas et al. also described EBUS findings related to chronic thromboembolic pulmonary hypertension as thrombus lining the vessel wall, organized intravascular thrombus, and intravascular fibrotic web ( 44 ).

One notable limitation of EBUS is its limited capacity to examine lesions located near the central airway. Additionally, the image quality is often suboptimal due to the lower frequency of EBUS ( 6 , 32 ).

Therapeutic application of EBUS

In recent decades, there has been a significant advancement in our understanding of lung cancer, leading to an expanded range of treatment options. Intratumoral therapy presents a viable approach in these circumstances, as it has promise in circumventing checkpoint resistance and reducing systemic side effects ( 45 ). The volume of studies investigating the use of chemotherapy and immunotherapy, or a combination of both, to target endobronchial or peribronchial lesions using EBUS is growing steadily ( 45 , 46 ). While EBUS enables precise real-time needle placement into the intended lesion, challenges remain regarding optimal positioning, necessary dosage, and the potential risk of drug retention ( 47 ).

EBUS is also employed for precise placement of fiducial markers, aiding in tracking for patients undergoing stereotactic radiation therapy for mediastinal and lung malignancies ( 48 , 49 ). In Majid et al. study, 51 fiducial markers were deployed in 37 patients via CP-EBUS guidance. Out of 37 patients, five necessitated a second procedure due to the unsuccessful placement of a fiducial marker. In this study, the median dimension of the lesion was recorded as 2.2 cm (IQR 0.4-3.3), and the median distance from the central airway was found to be 2.4 cm (IQR 0-3.4) ( 50 ). DeWitt and colleagues reported a retrospective study on endoscopic ultrasound guidance to drain pleural effusion. This study successfully utilized the EBUS scope in nine instances to insert into the esophagus and effectively drain pleural effusion. This procedure yielded sufficient samples for cytological investigation, demonstrating its efficacy without notable problems ( 51 ). Similarly, Cocciardi et al. conducted drainage of a loculated pleural effusion that could not be addressed through conventional thoracentesis ( 52 ). Additionally, aspiration of posteriorly loculated pericardial effusion via endobronchial ultrasound-guided approach was described in different case reports ( 53 , 54 ).

Other applications

EBUS has also been employed to sample pleural and pericardial lesions beyond effusions. Kassirer et al. reported a case that underwent pleural biopsy using EBUS with an esophageal approach ( 55 ). Hossain et al. described a patient in whom EBUS was utilized to diagnose pericardial mass ( 56 ). In previous reports, EBUS was used to diagnose thyroid nodules ( 57 , 58 ). Casal et al. documented 12 patients in which thyroid biopsies were conducted via CP-EBUS TBNA ( 59 ). The utilization of EBUS also provides microbiological assessment in pulmonary diseases. EBUS-TBNA is a diagnostic technique that effectively identifies pulmonary TB and tuberculosis lymphadenitis. The acquisition of sufficient lymphocytic material is beneficial for testing for acid-fast bacilli through staining and subsequent examination to determine the presence of necrotizing granulomas ( 60 , 61 ). A meta-analysis including eight studies with 809 patients revealed that the combined sensitivity and specificity values for diagnosing intrathoracic tuberculosis using EBUS-TBNA were 0.80 and 1.00, respectively. Still, the pooled sensitivity was more remarkable, at 0.87 ( 62 ). Using EBUS also enables obtaining material for fungal culture, so prompt detection of fungal infections such as histoplasmosis, mucormycosis, and coccidioidomycosis ( 63 , 64 , 65 , 66 ).

In summary, CP-EBUS serves to evaluate mediastinal lesions, provide guidance for needle aspiration, and explore the neighboring vascular structures close to the airway. Furthermore, it holds potential for therapeutic applications for pulmonary diseases. The increasing demand for tissue samples for diagnostic assessment and the expanding understanding of the pathological and molecular characteristics of lung cancer will further propel the advancement of the endobronchial ultrasound scope as a pivotal diagnostic tool in diverse pulmonary conditions.

## References

[bb0001] Yang H., Zhang Y., Wang K.P., Ma Y. (2015). Transbronchial needle
aspiration: Development history, current status and future
perspective. J Thorac Dis.

[bb0002] Biondini D., Tine M., Semenzato U., Daverio M., Scalvenzi F., Bazzan E. (13). Clinical applications of endobronchial
ultrasound (EBUS) scope: Challenges and opportunities.
Diagnostics (Basel). 2023.

[bb0003] Silvestri G.A., Gonzalez A.V., Jantz M.A., Margolis M.L., Gould M.K., Tanoue L.T. (2013). Methods for staging non-small cell
lung cancer: Diagnosis and management of lung cancer,
3rd ed: American College of Chest Physicians evidence-based clinical practice guidelines. Chest.

[bb0004] Vilmann P., Clementsen P.F., Colella S., Siemsen M., De Leyn P., Dumonceau J.M. (2015). Combined endobronchial and
oesophageal endosonography for the diagnosis and
staging of lung cancer. European Society of
Gastrointestinal Endoscopy (ESGE) Guideline, in
cooperation with the European Respiratory Society
(ERS) and the European Society of Thoracic Surgeons
(ESTS). Eur Respir J.

[bb0005] Wu J., Wu C., Zhou C., Zheng W., Li P. (2021). Recent advances in
convex probe endobronchial ultrasound: a narrative
review. Ann Transl Med.

[bb0006] Li P., Zheng W., Zhao L. (2015). Convex probe endobronchial ultrasound: Applications beyond conventional indications. J Thorac Dis.

[bb0007] Maturu V.N., Prasad V.P., Vaddepally C.R., Dommata R.R., Sethi S. (2023). Endobronchial ultrasound-guided mediastinal lymph
nodal cryobiopsy in patients with nondiagnostic/inadequate
rapid on-site evaluation: A new step in the diagnostic algorithm. J Bronchology Interv Pulmonol.

[bb0008] Erer O.F., Erol S., Anar C., Aydogdu Z., Ozkan S.A. (2017). Diagnostic
yield of EBUS-TBNA for lymphoma and review of the literature. Endosc Ultrasound.

[bb0009] Trisolini R., Lazzari Agli L., Tinelli C., De Silvestri A., Scotti V., Patelli M. (2015). Endobronchial ultrasound-guided transbronchial needle aspiration for diagnosis of sarcoidosis in clinically unselected study populations. Respirology.

[bb0010] Agarwal R., Srinivasan A., Aggarwal A.N., Gupta D. (2012). Efficacy
and safety of convex probe EBUS-TBNA in sarcoidosis: A
systematic review and meta-analysis. Respir Med.

[bb0011] Wang Y., Zhao Z., Zhu M., Zhu Q., Yang Z., Chen L. (2023). Diagnostic value of endobronchial ultrasound elastography in differentiating between benign and malignant hilar
and mediastinal lymph nodes: A retrospective study. Quant Imaging Med Surg.

[bb0012] Zhang J., Guo J.R., Huang Z.S., Fu W.L., Wu X.L., Wu N. (2021). Transbronchial mediastinal cryobiopsy in the diagnosis of
mediastinal lesions: A randomised trial.. Eur Respir J.

[bb0013] Ariza-Prota M.A., Perez-Pallares J., Fernandez-Fernandez A., Lopez-Gonzalez F., Cascon J.A., Garcia-Alfonso L. (2022). Transbronchial mediastinal cryobiopsy in the diagnosis of
mediastinal lymph nodes: A case series - How to do it. Arch Bronconeumol.

[bb0014] Oikonomidou R., Petridis D., Kosmidis C., Sapalidis K., Hohenforst-Schmidt W., Christakidis V. (2022). Cryo-Biopsy
versus 19G needle versus 22G needle with EBUS-TBNA
endoscopy. J Cancer.

[bb0015] Annema J.T., Rozman A. (2023). Endobronchial ultrasound-guided
cryobiopsy-when it is indicated?. Lancet Respir Med.

[bb0016] Fan Y., Zhang A.M., Wu X.L., Huang Z.S., Kontogianni K., Sun K. (2023). Transbronchial needle aspiration combined with
cryobiopsy in the diagnosis of mediastinal diseases: A
multicentre, open-label, randomised trial. Lancet Respir Med.

[bb0017] Ariza-Prota M., Perez-Pallares J., Fernandez-Fernandez A., Garcia-Alfonso L., Cascon J.A., Torres-Rivas H. (2023). Endobronchial ultrasound-guided transbronchial mediastinal cryobiopsy in the diagnosis of mediastinal lesions:
Safety, feasibility and diagnostic yield - experience in 50
cases. ERJ Open Res.

[bb0018] Darwiche K., Freitag L., Nair A., Neumann C., Karpf-Wissel R., Welter S. (2013). Evaluation of a novel endobronchial ultrasound-guided lymph node forceps in enlarged mediastinal
lymph nodes. Respiration.

[bb0019] Herth F.J., Schuler H., Gompelmann D., Kahn N., Gasparini S., Ernst A. (2012). Endobronchial ultrasound-guided lymph
node biopsy with transbronchial needle forceps: A pilot
study. Eur Respir J.

[bb0020] Cheng G., Mahajan A., Oh S., Benzaquen S., Chen A. (2019). Endobronchial ultrasound-guided intranodal forceps biopsy (EBUS-IFB)-technical review. J Thorac Dis.

[bb0021] Agrawal A., Ghori U., Chaddha U., Murgu S. (2022). Combined
EBUS-IFB and EBUS-TBNA vs. EBUS-TBNA alone for intrathoracic adenopathy: A meta-analysis.. Ann Thorac Surg.

[bb0022] Balwan A., Bixby B., Grotepas C., Witt B.L., Iravani A., Ansari S. (2020). Core needle biopsy with endobronchial ultrasonography: Single center experience with 100 cases. J Am Soc Cytopathol.

[bb0023] Pausawasdi N., Cheirsilpa K., Chalermwai W., Asokan I., Sriprayoon T., Charatcharoenwitthaya P. (2022). Endoscopic ultrasound-guided fine-needle biopsy using 22G Franseen
needles without rapid on-site evaluation for diagnosis of
intraabdominal masses. J Clin Med.

[bb0024] Dhooria S., Sehgal I.S., Prasad K.T., Muthu V., Gupta N., Bal A. (2021). Diagnostic yield and safety of the ProCore versus the
standard EBUS-TBNA needle in subjects with suspected
sarcoidosis. Expert Rev Med Devices.

[bb0025] Yang L., Gu Y., Wang H., Yu D., Zhang H., Wang H. (2020). Novel
ProCore 25-gauge needle for endobronchial ultrasound-guided transbronchial needle aspiration reduces
the puncture time and frequency, with comparable diagnostic rate for mediastinal and hilar lymphadenopathy. Thorac Cancer.

[bb0026] Karsenti D., Palazzo L., Perrot B., Zago J., Lemaistre A.I., Cros J. (2020). 22G Acquire vs. 20G procore needle for endoscopic
ultrasound-guided biopsy of pancreatic masses: A
randomized study comparing histologic sample quantity
and diagnostic accuracy. Endoscopy.

[bb0027] Wang B., Guo Q., Wang J.Y., Yu Y., Yi A.J., Cui X.W. (2021). Ultrasound elastography for the evaluation of lymph
nodes. Front Oncol.

[bb0028] Dietrich C.F., Jenssen C., Herth F.J. (2016). Endobronchial ultrasound elastography. Endosc Ultrasound.

[bb0029] Wu J., Sun Y., Wang Y., Ge L., Jin Y., Wang Z. (2022). Diagnostic value
of endobronchial ultrasound elastography for differentiating
benign and malignant hilar and mediastinal lymph
nodes: A systematic review and meta-analysis. Med Ultrason.

[bb0030] Madan M., Mittal S., Tiwari P., Hadda V., Mohan A., Guleria R. (2022). The diagnostic utility of ultrasound elastography
to differentiate tuberculosis and sarcoidosis during endobronchial ultrasound-guided transbronchial needle aspiration (EBUS-TBNA). Lung India.

[bb0031] Al-Saffar F., Ibrahim S., Seeram V., Bajwa A.A., Shujaat A. (2015). Use
of endobronchial ultrasound to evaluate nonthrombotic
endovascular lesions in pulmonary arteries: A systematic
review.. J Bronchology Interv Pulmonol.

[bb0032] Aljohaney A.A. (2019). Role of convex probe endobronchial ultrasound in the diagnosis and treatment of nonmalignant
diseases. Pulm Med.

[bb0033] Casoni G.L., Gurioli C., Romagnoli M., Poletti V. (2008). Diagnosis of
pulmonary thromboembolism with endobronchial ultrasound. Eur Respir J.

[bb0034] Swartz M.A., Gillespie C.T. (2011). Pulmonary emboli detected by
endobronchial ultrasound. Am J Respir Crit Care Med.

[bb0035] Egea Santaolalla C.J., Ribas Solis F.J., Juste Carne M. (2011). Pulmonary thromboembolism observed by endobronchial
ultrasound (EBUS). Arch Bronconeumol.

[bb0036] Llopis Pastor E., Franco Serrano J., Bures Sales E. (2013). Pulmonary
thromboembolism diagnosed by endobronchial ultrasound. Arch Bronconeumol.

[bb0037] Sachdeva A., Lee H.J., Malhotra R., Shepherd R.W. (2013). Endobronchial ultrasound diagnosis of pulmonary embolism. J Bronchology Interv Pulmonol.

[bb0038] Chow J., Roberts J., Trivedi K. (2021). Utility of bronchoscopy with
endobronchial ultrasound in diagnosis and monitoring of
pulmonary embolism. Cureus.

[bb0039] Li P., Wu C., Zheng W., Zhao L. (2017). Pathway and application
value of exploration of the pulmonary artery by endobronchial ultrasound.. J Thorac Dis.

[bb0040] Aumiller J., Herth F.J., Krasnik M., Eberhardt R. (2009). Endobronchial
ultrasound for detecting central pulmonary emboli: A
pilot study. Respiration.

[bb0041] Juul A.D., Laursen C.B., Christophersen A., Arshad A., Luef S.M., Panou V. (2023). Endobronchial ultrasound for the screening
of pulmonary embolism in patients with suspected lung
cancer: A prospective cohort study. Respiration.

[bb0042] Chauhan N.K., Rajagopal R., Shadrach B.J., Dutt N. (2023). Pulmonary
arterio-venous malformation on endobronchial ultrasound
bronchoscopy. Thorax.

[bb0043] Sanz-Santos J., Andreo F., Garcia-Olive I., Remon J., Monso E. (2011). Diagnosis of acute pulmonary embolism by endobronchial ultrasound as an incidental finding. Respiration.

[bb0044] Ghattas C., Channick R.N., Wright C.D., Vlahakes G.J., Channick C. (2021). Vascular endobronchial ultrasound in a
patient with chronic thromboembolic pulmonary hypertension. J Bronchology Interv Pulmonol.

[bb0045] Mori v., Bates J.H.T., Jantz M., Mehta H.J., Kinsey C.M. (2022). A computational modeling approach for dosing endoscopic
intratumoral chemotherapy for advanced non-small cell
lung cancer. Sci Rep.

[bb0046] Zarogoulidis P., Hohenforst-Schmidt W., Huang H., Zhou J., Wang Q., Wang X. (2021). Intratumoral treatment with chemotherapy and immunotherapy for NSCLC with EBUSTBNA 19G. J Cancer.

[bb0047] Mori V., DuComb E.A., Wagner S., Khan F., Bates J.H.T., Kinsey C.M. (2023). Visualizing intratumoral injections in lung tumors by
endobronchial ultrasound. J Cancer.

[bb0048] Seides B.J., Egan J.P. 3rd, French K.D., Kovitz K.L., Desai N.R. (2018). Fiducial marker placement for stereotactic body radiation
therapy via convex probe endobronchial ultrasound: A
case series and review of literature. J Thorac Dis.

[bb0049] Casutt A., Kinj R., Ozsahin E.M., von Garnier C., Lovis A. (2022). Fiducial markers for stereotactic lung radiation therapy:
Review of the transthoracic, endovascular and endobronchial approaches. Eur Respir Rev.

[bb0050] Majid A., Palkar A., Kheir F., Alape D., Fernandez-Bussy S., Aronovitz J. (2018). Convex probe EBUS-guided fiducial
placement for malignant central lung lesions.. J Bronchology Interv Pulmonol.

[bb0051] DeWitt J., Kongkam P., Attasaranya S., LeBlanc J.K., Sherman S., Sheski F.D. (2008). Endoscopic ultrasound-guided transesophageal thoracentesis. Endoscopy.

[bb0052] Cocciardi S., Borah A., Terrigno R., Abouzgheib W., Boujaoude Z. (2019). A case report of an expensive yet necessary
thoracentesis: Expanding the boundaries of endoscopic
ultrasound transbronchial needle aspiration. Medicine
(Baltimore).

[bb0053] Sharma R.K., Khanna A., Talwar D. (2016). Endobronchial ultrasound: A new technique of pericardiocentesis in posterior
loculated pericardial effusion. Chest.

[bb0054] Christiansen I.S., Clementsen P.F., Petersen J.K., Fjaellegaard K., Hoegholm A., Bodtger U. (2020). Aspiration of pericardial effusion performed with EUS-B-FNA in suspected lung cancer. Respiration.

[bb0055] Kassirer M., Wiesen J., Atlan K., Avriel A. (2018). Sampling pleural
nodules with an EBUS scope: A novel application. Med Case Rep.

[bb0056] Hossain S., Chawla M. (2020). Traversing uncharted territory:
Endobronchial ultrasound utilized for diagnosis of pericardial mass. Chest.

[bb0057] Chalhoub M., Harris K. (2012). Endobronchial ultrasonography
with transbronchial needle aspiration to sample a solitary
substernal thyroid nodule: A new approach. Heart Lung Circ.

[bb0058] Chalhoub M., Harris K. (2010). The use of endobronchial ultrasonography with transbronchial needle aspiration to sample
a solitary substernal thyroid nodule. Chest.

[bb0059] Casal R.F., Phan M.N., Keshava K., Garcia J.M., Grosu H., Lazarus D.R. (2014). The use of endobronchial ultrasound-guided transbronchial needle aspiration in the
diagnosis of thyroid lesions. BMC Endocr Disord.

[bb0060] Goussard P., Eber E., Venkatakrishna S., Frigati L., Janson J., Schubert P. (2023). Intrathoracic tuberculosis: Role of interventional bronchoscopy in diagnosis. Paediatr Respir Rev.

[bb0061] Cong C.V., Ly T.T., Anh P.Q., Duc N.M. (2022). Primary mediastinal
lymph node tuberculosis diagnosed using endobronchial
ultrasound-guided transbronchial needle aspiration:
Literature review and case report. Radiol Case Rep.

[bb0062] Ye W., Zhang R., Xu X., Liu Y., Ying K. (2015). Diagnostic efficacy and
safety of endobronchial ultrasound-guided transbronchial
needle aspiration in intrathoracic tuberculosis: A
meta-analysis. J Ultrasound Med.

[bb0063] Egressy K., Mohammed M., Ferguson J.S. (2015). The use of endobronchial ultrasound in the diagnosis of subacute pulmonary histoplasmosis. Diagn Ther Endosc.

[bb0064] Nair V., Sharma R.K., Khanna A., Talwar D. (2017). Pulmonary
mucormycosis diagnosed by convex probe endobronchial
ultrasound-guided fine needle aspiration of cavity wall. Lung India.

[bb0065] Shah R.A., Vempilly J.J., Noor U.I., Husnain S.M., Hegde P. (2018). Combined endosonography reduces time to diagnose
pulmonary coccidioidomycosis. J Bronchology Interv Pulmonol.

[bb0066] Fernandez-Arias C., Barrio-Herraiz E., Centeno-Clemente C., Garcia-Olive I., Tazi-Mezalek R., Andreo-Garcia F. (2023). Usefulness of routine bronchial aspirate of endobronchial
ultrasound-guided transbronchial needle aspiration. Arch Bronconeumol.

